# Criteria for detection of possible risk factors for mental health problems in undergraduate university students

**DOI:** 10.3389/fpsyt.2023.1184156

**Published:** 2023-06-29

**Authors:** Daiki Ishimaru, Hiroyoshi Adachi, Teruhiro Mizumoto, Viktor Erdelyi, Hajime Nagahara, Shizuka Shirai, Haruo Takemura, Noriko Takemura, Mehrasa Alizadeh, Teruo Higashino, Yasushi Yagi, Manabu Ikeda

**Affiliations:** ^1^Department of Psychiatry, Osaka University Graduate School of Medicine, Osaka, Japan; ^2^Health and Counseling Center, Osaka University, Osaka, Japan; ^3^Department of Information Networking, Osaka University Graduate School of Information Science and Technology, Osaka, Japan; ^4^Institute for Datability Science, Osaka University, Osaka, Japan; ^5^Infomedia Education Research Division, Cybermedia Center, Osaka University, Osaka, Japan; ^6^Department of Artificial Intelligence, Graduate School of Computer Science and Systems Engineering, Kyushu Institute of Technology, Fukuoka, Japan; ^7^Department of Information Technology, Faculty of Technology, International Professional University of Technology, Osaka, Japan; ^8^The Institute of Scientific and Industrial Research, Osaka, Japan

**Keywords:** early detection, lifestyle habit, mental health, study engagement, university students

## Abstract

**Introduction:**

Developing approaches for early detection of possible risk clusters for mental health problems among undergraduate university students is warranted to reduce the duration of untreated illness (DUI). However, little is known about indicators of need for care by others. Herein, we aimed to clarify the specific value of study engagement and lifestyle habit variables in predicting potentially high-risk cluster of mental health problems among undergraduate university students.

**Methods:**

This cross-sectional study used a web-based demographic questionnaire [the Utrecht Work Engagement Scale for Students (UWES-S-J)] as study engagement scale. Moreover, information regarding life habits such as sleep duration and meal frequency, along with mental health problems such as depression and fatigue were also collected. Students with both mental health problems were classified as high risk. Characteristics of students in the two groups were compared. Univariate logistic regression was performed to identify predictors of membership. Receiver Operating Characteristic (ROC) curve was used to clarify the specific values that differentiated the groups in terms of significant predictors in univariate logistic analysis. Cut-off point was calculated using Youden index. Statistical significance was set at *p* < 0.05.

**Results:**

A total of 1,644 students were assessed, and 30.1% were classified as high-risk for mental health problems. Significant differences were found between the two groups in terms of sex, age, study engagement, weekday sleep duration, and meal frequency. In the ROC curve, students who had lower study engagement with UWES-S-J score < 37.5 points (sensitivity, 81.5%; specificity, 38.0%), <6 h sleep duration on weekdays (sensitivity, 82.0%; specificity, 24.0%), and < 2.5 times of meals per day (sensitivity, 73.3%; specificity, 35.8%), were more likely to be classified into the high-risk group for mental health problems.

**Conclusion:**

Academic staff should detect students who meet these criteria at the earliest and provide mental health support to reduce DUI among undergraduate university students.

## 1. Introduction

Mental health problems among university students are very common and result in underutilization of college services. Adolescence and young adulthood, which generally corresponds with the age of undergraduate university students, have been reported to be the peak ages for onset of mental disorders, such as mood disorders or addictive behaviors (1). Additionally, a recent systematic review focusing on the mental health problems of undergraduate university students reported that the pooled prevalence of depression and suicide-related issues were 25 and 14%, respectively (2). Early intervention or prevention of mental health problems is therefore required, especially among undergraduate university students.

Recently, duration of untreated illness (DUI), which is the time span between the onset of a psychiatric symptom and the first appropriate treatment (3), has garnered attention as one of the most significant concerns in mental health problems among undergraduate university students. DUI has been known to have a critical impact on treatment response, symptom control, and clinical course (3, 4). It has been reported that among the university students who first visited the psychiatry department of health care centre, 48.2 and 36.7% took more than 6 months and 1 year, respectively, to consulting a psychiatrist (5), emphasizing delayed psychiatric help-seeking behavior in undergraduate university students. Several factors such as poor general health condition, absence of physical symptoms, or stigma of mental health problems have been suggested to affect the delayed help-seeking behavior in a complex way (5–7). Therefore, more attention should be paid to develop approaches for not only increasing their help-seeking behavior, but also in detecting the potential risk cluster of mental health problems at the earliest.

Numerous factors have been indicated to correlate to mental health problems among university students (8, 9). One study (8) categorized the influencing factors for depression among university students into the following four aspects: biological factors, personality and psychological state, college experience, and lifestyle. However, many university students tend to avoid disclosing their mental health state or personal details due to embarrassment or stigma (10, 11). Considering these findings, psychological and personal assessment might not be easily accessible in the usual academic practice, although such information is crucial to managing mental health problems. Therefore, it could be useful to focus on the indirect and accessible risk factors such as college experience or lifestyle to better identify and support the potential risk cluster of mental health problems. For example, a previous study reported that university students with lower study engagement were likely to have several school life crises, including leaving school or taking academic years off, and suggested the necessity of intensive psychological or psychiatric support for such students (12). In other studies, sleep disturbances were found to be a more common problems among students with various mental health disorders and to increase the risk for the subsequent onset of major depression (13, 14). Additionally, poor eating habits are considered as one of the lifestyle risk factors for several mental health problems among university students (15). Although these factors might be relatively accessible as risk factors for mental health problems in the usual academic setting, little evidence is available regarding the specific or indicative values of these variables, which could induce the subsequent development of mental health problems or reflect the secondary impact from these conditions. Thus, academic staff members have been refractory to early detection of students with potential mental health problems among populations with delayed help-seeking behavior and preventing their condition from worsening, since the criteria of decreased study engagement or lifestyle habit problems remain ambiguous.

Therefore, this study aimed to clarify the specific value of study engagement and lifestyle habit variables in predicting potentially high-risk clusters of mental health problems among undergraduate university students.

## 2. Materials and methods

### 2.1. Study design

This cross-sectional observational study was a part of the survey which sought to investigate the contents and features of online education provided to students enrolled at Osaka University during the novel coronavirus disease 2019 (COVID-19) pandemic, while also focusing on the characteristics of school adaptation and their mental health (16).

The survey used an online questionnaire to assess all the online classes and mental health parameters of the participants. The study tool was distributed among participants *via* the online education support system of Osaka University; students accessed this tool using their own personal computers, tablets, or smartphones. Information regarding all the students of Osaka University was registered in the online education support system. Students could not create multiple identities to answer the questionnaire. The questionnaire included a written explanation that completing the questionnaire implied informed consent, that participation in the study was voluntary, and that no negative consequences would occur if they chose not to participate.

### 2.2. Study setting

This study was conducted at Osaka University, Osaka Prefecture, Japan, between July 27, 2020 and August 10, 2020.

### 2.3. Study participants

This study recruited undergraduate university students at Osaka University. Inclusion criteria were: (1) the students in first year of undergraduate study and (2) the regular affiliation students who were currently enrolled at Osaka University. However, students who had any of the following conditions were excluded: (1) temporarily absent or studying abroad and (2) having any missing assessment data.

**Figure 1:**
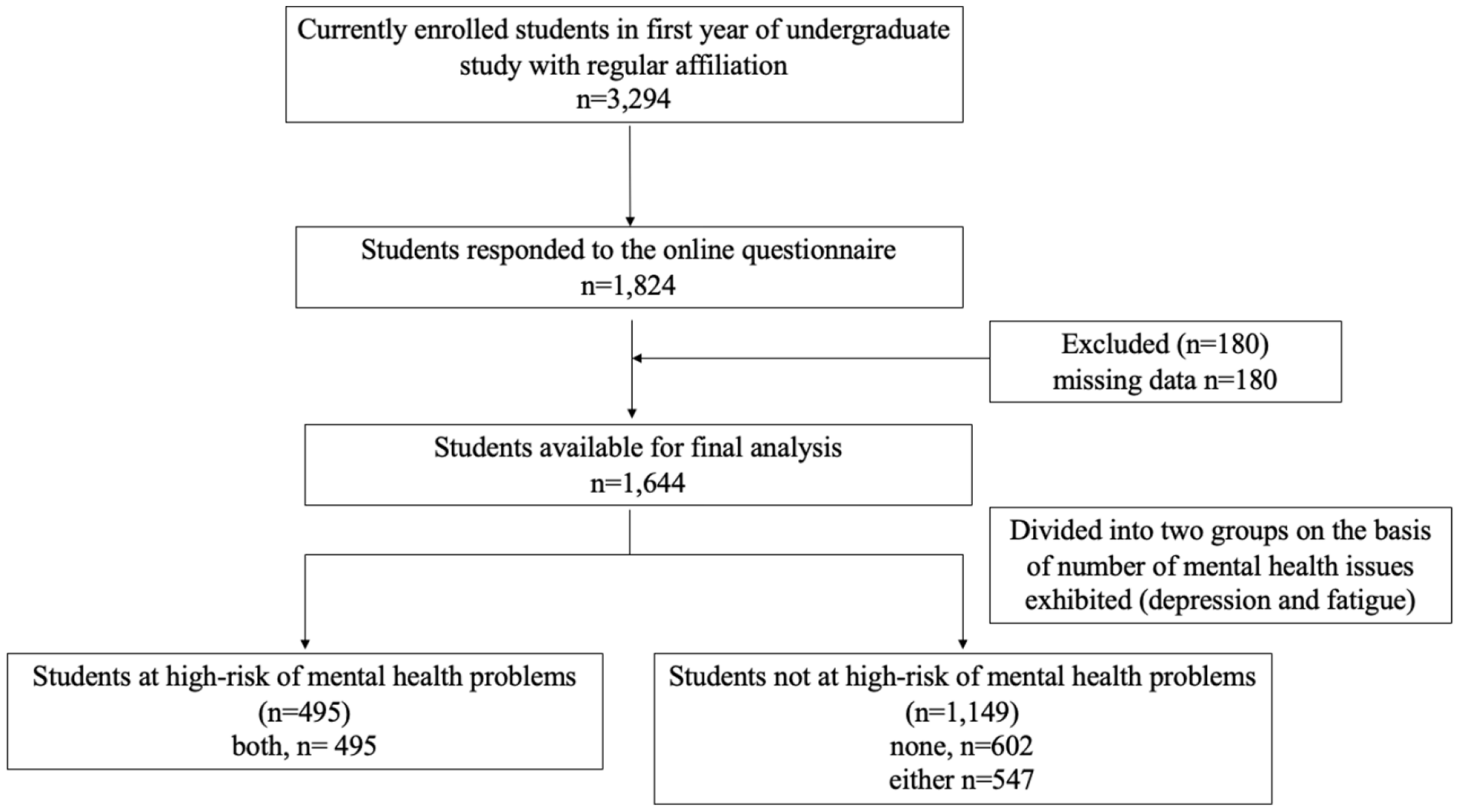
Flow chart of the study participants who met the inclusion/exclusion criteria.

A total of 15,194 undergraduate students were enrolled at Osaka University. First-year undergraduate students accounted for 22.4% of the total number of students. The online questionnaire was sent to 3,294 students, of which 1, 824 responded (55.4%). Based on the eligibility criteria, 1,644 students (49.9%) were included in the analysis (Figure 1).

### 2.4. Study variables

The details of the study parameters are presented under the respective variable sub-sections.

#### 2.4.1. Demographics

Demographic data of the participants were obtained using the Osaka University’s online education support system. Information regarding the following variables were collected: sex, age, and faculty of the respective classes.

#### 2.4.2. Study engagement

The Japanese version of the Utrecht Work Engagement Scale for Students (UWES-S-J) was used to assess study engagement (17). It consists of 14 items with three subdomains: vigour (five items), dedication (five items), and absorption (four items). The item examples of vigour are as follows: “When I’m studying, I feel mentally strong,” “I can continue for a very long time when I am studying,” or “When I study, I feel like I am bursting with energy,” The item examples of dedication are as follows: “I find my studies to be full of meaning and purpose,” “My studies inspire me,” or “I am enthusiastic about my studies.” The item examples of absorption are as follows: “Time flies when I’m studying.,” “When I am studying, I forget everything else around me,” or “I feel happy when I am studying intensively.” All items were rated on a 7-point scale (0 = never, 6 = always). Total scores ranged between 0 and 84, with higher scores indicating better study engagement. The terms of each question were modified to assess online education engagement. The UWES-S-J has been reported to show good reliability and validity in assessing study engagement among Japanese students (17).

#### 2.4.3. Mental health problems

Participants were asked to self-assess the presence of subjective depression and fatigue by answering the following two questions: “Do you feel depressed?” and ‘Do you feel tired?’. The responses to these questions could either be “Yes” or “No.”

#### 2.4.4. Lifestyle habits

Participants were asked to self-report their total sleep duration on weekdays and holidays during the past month by answering the following questions: “What are your sleeping hours on the weekdays?” and “What are your sleeping hours on weekends/holidays?.” The response to these questions could be “Less than 5 h,” “5–6 h,” “6–7 h,” “7–8 h,” “8–9 h,” and “9 h or more.” These scores were replaced with approximate durations: 4.5 h, 5.5 h, 6.5 h, 7.5 h, 8.5 h, and 9.5 h, respectively.

The mean number of meals per day in the past month was assessed as follows: “How many meals do you eat a day?” The response to these questions were “There are days when I do not eat,” “Once,” “Twice,” “Three times,” and “Four or more times.” The score of “Four or more times” was replaced with four times per day in the statistical analysis.

### 2.5. Statistical analysis

Descriptive statistics were calculated for all the variables. Normal distribution was checked using a Q-Q plot and histogram. Equality of error variances for variables was checked using Levine’s test.

Participants were divided into two groups based on their mental health status. Students who exhibited both depression and fatigue were classified as high-risk mental health group, while all the others were incorporated in the non-high-risk mental health group.

Cohen’s *d* with Student’s t test/Welch *t* test, *r* values with Mann–Whitney *U* test, and *φ* value with Pearson’s chi-square test were used to examine differences in the demographics, study engagement, and lifestyle habits between the two groups, respectively. In the effect size of Mann–Whitney, *r* was calculated as *Z*/√n. Additionally, univariable logistic regression analysis was performed to identify predictors of group membership with high-risk of mental health problems. Group membership of mental health state (high-risk group vs. non-high-risk group) was included as a dependent variable. Study engagement, weekday sleep duration, holiday sleep duration, and number of meals per day were included as independent variables.

A receiver operating characteristic (ROC) curve was also used to clarify the specific values that differentiated the group with high-risk of mental health problems from the group without high-risk of mental health problems. Variables with significant predictors in univariate logistic analysis were used in this test. The cut-off point was calculated with sensitivity and specificity using the Youden index.

All analyses were conducted using SPSS 28 (IBM Inc., Chicago, IL, United States). A *p* value of <0.05 was considered statistically significant.

### 2.6. Ethical considerations

All procedures contributing to this study complied with the ethical standards of the relevant national and institutional committees on human experimentation and the tenets of Declaration of Helsinki. All procedures involving human participants were approved by the Ethics Committee of the Institute for Datability Science of Osaka University (July 21, 2020). Informed consent was obtained from all the study participants.

## 3. Results

### 3.1. Characteristics of the participants and comparison between the two groups

Table 1 shows the characteristics of the 1,644 participants. Most participants were male students (65.3%). The age of the study participants ranged between 18–25 years, and the mean age was 18.69 (0.80) years. Among the study population, 1,009 participants (61.4%) belonged to the science stream, while the remaining 635 (38.6%) belonged to the humanities stream.

**Table 1:** Participants characteristics and group classification.

Variables	Whole sample *n* = 1,644	Group with high-risk of mental health problems *n* = 495	Group without high-risk of mental health problems *n* = 1,149	*p* value	Effect size
Sex, *n* (%)					
Male	1,073 (65.3%)	304 (61.4%)	769 (66.9%)		
Female	571 (34.7%)	191 (38.6%)	380 (33.1%)	0.031^a^	0.053
Age, mean years (SD)	18.69 (0.80)	18.77 (0.84)	18.65 (0.78)	0.003^b^	0.072
Stream, *n* (%)					
Science	1,009 (61.4%)	308 (62.2%)	701 (61.0%)		
Humanities	635 (38.6%)	187 (37.8%)	448 (39.0%)	0.643^a^	
UWES-S-J	45.93 (13.90)	41.26 (14.38)	47.93 (13.20)	<0.001^c^	0.492
Sleep duration (h), mean (SD)					
Sleep duration on weekdays	6.81 (1.00)	6.69 (1.06)	6.86 (0.97)	0.002^d^	
Sleep duration on holidays	7.44 (1.08)	7.40 (1.16)	7.45 (1.04)	0.365^c^	0.168
Food, mean (SD)					
Number of meals per day	2.70 (0.56)	2.61 (0.63)	2.74 (0.53)	<0.001^b^	0.092

UWES-S-J, Japanese version of the Utrecht Work Engagement Scale for Students; SD, standard deviation.

a φvalue with Pearson’s chi-square test.

b *r* value with Mann–Whitney *U* test.

c Cohen’s d with Welch *t* test.

d Cohen’s d with Student *t* test.

A total of 495 participants (30.1%) were classified into the group with high-risk of mental health problems, while the remaining 1,213 (69.9%) were allocated into the group without high-risk of mental health problems. In the latter group, 602 participants (36.7%) had no mental health problems, while 547 (33.2%) exhibited either depression or fatigue. Table 1 shows the comparison between the two groups. Significant differences were observed between the two groups in terms of sex, age, UWES-S-J scores, weekday sleep duration, and number of meals. The effect sizes for comparison of sex, age, weekday sleep duration, and number of meals per day were small (*φ* = 0.053, *r* = 0.072, Cohen’s *d* = 0.168, and *r* = 0.092, respectively), while that for UWES-S-J scores ranged between small and moderate (Cohen’s *d* = 0.492).

### 3.2. Logistic regression analysis

Table 2 shows the results of univariate regression analysis. Among the four independent variables, USES-S-J scores, weekday sleep duration, and number of meals per day were significantly associated with membership of the high-risk mental health group. Students with lower study engagement, shorter weekday sleep duration, and fewer meals per day were more likely to be classified into the high-risk mental health group.

**Table 2:** Univariable logistic regression analysis predicting the group with high-risk of mental health problems.

Variables	B	Odds ratio	95% confidence interval		*p* value
			Lower	Upper	
UWES-S-J	−0.36	0.964	0.956	0.972	<0.001
Sleep duration on weekdays	−0.169	0.844	0.759	0.939	0.002
Sleep duration on holidays	−0.047	0.954	0.865	1.052	0.343
Number of meals per day	−0.372	0.689	0.574	0.828	<0.001

UWES-S-J, Japanese version of the Utrecht Work Engagement Scale for Students.

### 3.3. ROC analysis

**Figure 2:**
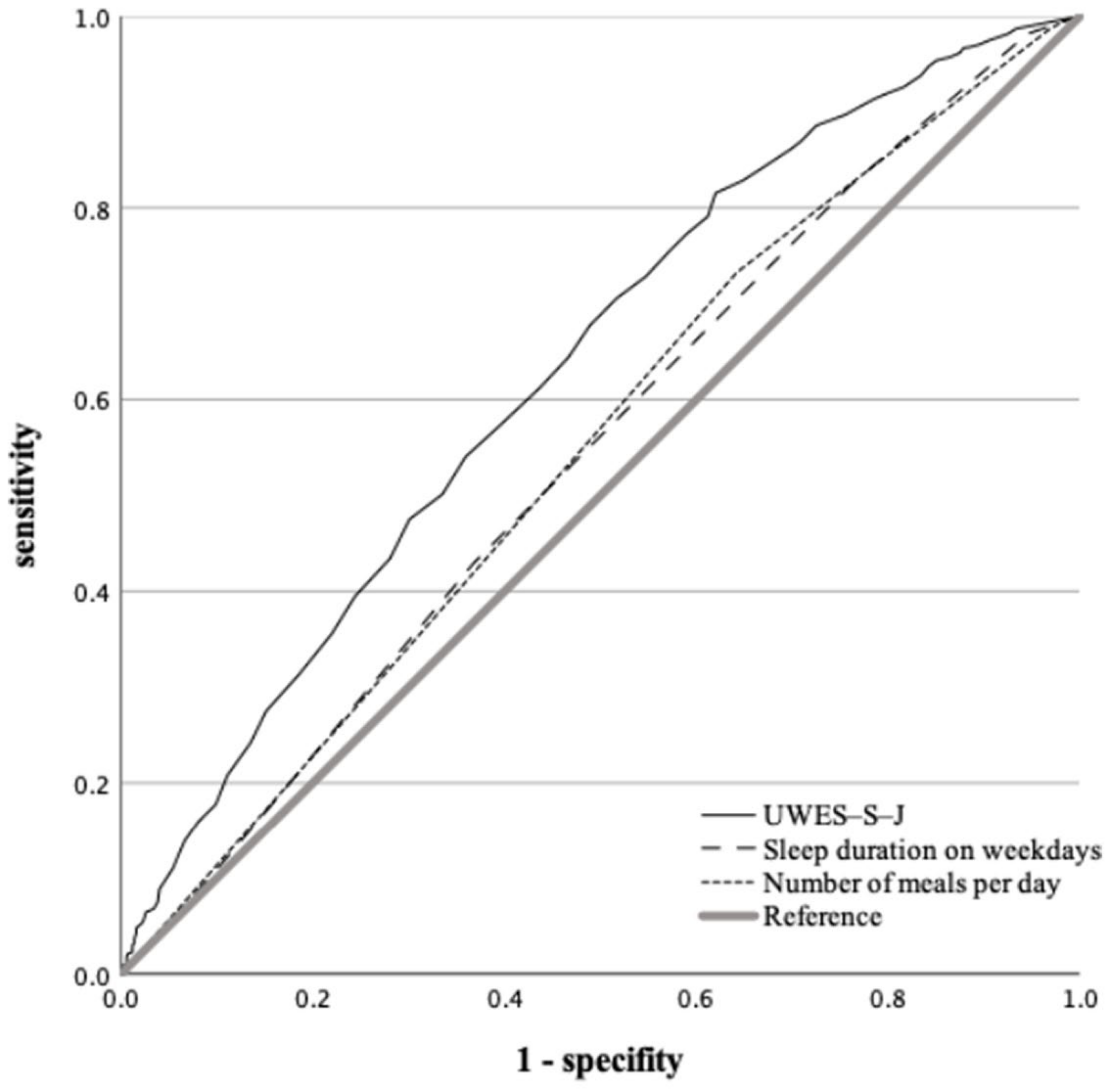
Receiver Operating Characteristic curve differentiating between the group with high-risk of mental health problems from group without high-risk of mental health problems. The AUC for UEWS-S-J score was 0.631 (*p* < 0.001; 95% confidence interval, 0.602–0.661) with cut-off point of <37.5 (sensitivity, 81.5%; specificity, 38.0%). The AUC for weekday sleep duration was 0.544 (*p* = 0.004; 95% confidence interval, 0.513–0.575) with cut-off point of <6 h (sensitivity, 82.0%; specificity, 24.0%). The AUC for number of meals per day was 0.547 (*p* = 0.003; 95% confidence interval, 0.516–0.578) with cut-off point of <2.5 times/day (sensitivity, 73.3%; specificity, 35.8%). AUC, area under the curve; UWES-S-J, Japanese version of the Utrecht Work Engagement Scale for Students.

In the ROC analysis, we used the following three variables: UWES-S-J scores, weekday sleep duration, and number of meals per day, as they were significant predictors in the univariate logistic regression analysis. Figure 2 shows the ROC curves of the variables that differentiated the two groups.

The areas under the curve (AUC) for UEWS-S-J scores, weekday sleep duration, and number of meals per day were 0.631 (*p* < 0.001; 95% confidence interval, 0.602–0.661), 0.544(*p* = 0.004; 95% confidence interval, 0.513–0.575), and 0.547 (*p* = 0.003; 95% confidence interval, 0.516–0.578), respectively.

The optimal cut-off points for UEWS-S-J scores, weekday sleep duration, and number of meals per day were < 37.5 points (sensitivity, 81.5%; specificity, 38.0%), < 6 h (sensitivity, 82.0%; specificity, 24.0%), < 2.5 times/day (sensitivity, 73.3%; specificity, 35.8%), respectively.

## 4. Discussion

This study clarified the specific value of study engagement and lifestyle habits, such as sleep or meal frequency, that could predict the group at potentially high risk for mental health problems in a large sample of first-year undergraduate university students. We yielded two major findings. First, approximately 30% of the first-year undergraduate university students were at a high risk of mental health problems, although no diagnosis or support had been provided to the majority of them. Second, students who had lower study engagement with UWES-S-J score < 37.5, less than 6 h of weekday sleep duration, and less than 2.5 times of meals per day were more likely to be classified into the group with high-risk of mental health problems in ROC curve analysis.

We classified the participants into two groups based on subjective depression and fatigue. These states were diagnosed in accordance with the Major Depression Disorders (MDD) in Diagnostic and Statistical Manual of Mental Disorders, Fifth Edition (DSM-5) (18), although their severity and objectivity were not confirmed. Depression is a common mental health problem among undergraduate university students (19). A state that meets the diagnostic criteria of MDD is highly suggestive of high risk of mental health crisis. Our results regarding the prevalence of mental health problems were mostly in line with those of previous systematic reviews, which reported that the pooled prevalence of depression was 25%, and ranged between 10–58%, among undergraduate university students (2). However, the present study was conducted during the COVID-19 pandemic, which may suggest the possibility that the number of students with mental health problems had increased during this period in comparison to the usual conditions (20, 21).

To the best of our knowledge, this is the first study to have identified specific values of study engagement and lifestyle habits that could differentiate between groups with and without high-risk of mental health problems. Several studies have assessed study engagement using the UWES-S scores (22–25), however, none of them have demonstrated cut-off point of UWES-S score for mental health problems. Moreover, these studies have inconsistently used the unified version of the UWES-S, which were prepared in the following three versions: 9, 14, and 17 items (26, 27). These factors would make it difficult to compare the mean score of the UWES-S in our sample and the cut-off point with previous results. However, a score of 37.5/98 (acquisition rate of 38.3%) might serve as an index for the academic staff to pay attention towards mental health support needs.

A previous systematic review reported a moderate association between insomnia and psychological stress among undergraduate university students, and also suggested that the relationship was bidirectional in nature (28). Notably, weekday sleep duration was found to be significantly associated with mental health problem in the present study. This could be partially explained by the interpretation that psychological stress has a greater impact on sleep on weekdays compared to holidays in undergraduate university students. Decrease in meal frequency has been associated with depression and anxiety among university students (29). Furthermore, skipping meals, especially breakfast, has been suggested to be a risk factor for depression among undergraduate university students (30). Taken together, meal frequency < 2.5 times/day could help identify students with potential mental health problems. However, the present study could not assess which meal the participants skipped.

This study had a few limitations. First, this study defined high risk of mental health problems based on subjective depression and fatigue, although these symptoms were based on the criteria for MDD in the DSM-5. The symptoms would need to be correctly diagnosed by an expert psychiatrist. A validated scale that can assess a comprehensive state, such as anxiety or stress, should also be employed to accurately evaluate the risk of mental health problems. Second, specific values for predicting potential groups with high-risk of mental health problems were calculated using a cross-sectional design. Temporal changes in study engagement and lifestyle habits are needed to further investigate the relationships with occurrence of mental health problems. Third, the present study did not include other predictors, such as economic situation or interaction with friends. This limitation highlights the need to consider the potential predictors of mental health problems. Finally, the specificity of cut-offs in the ROC curve analysis was relatively low, although the sensitivity ranged between 73.3–82.0%. However, for early detection, it may be important to avoid overlooking students with potential mental health problems, emphasizing the establishment of good sensitivity rather than specificity.

## 5. Conclusion

In conclusion, this study found that students who had lower study engagement with UWES-S-J score < 37.5, less than 6 hours of weekday sleep duration, and less than 2.5 times of meals per day were more likely to be classified into the group with high-risk of mental health problems. Academic staff should detect students who meet these criteria at the earliest and provide mental health support to reduce DUI among undergraduate university students. We believe that a sensing technology which could automatically obtain these assessments might be useful in outreach for individuals with potential mental health problems. Future studies should investigate the specific value of temporal changes in study engagement and lifestyle habit variables that could predict the occurrence of mental health problems among undergraduate university students.

## Data availability statement

The datasets presented in this article are not readily available because the datasets generated during the current study are not publicly available due to privacy considerations of the participants. Requests to access the datasets should be directed to HA hadachi@psy.med.osaka-u.ac.jp.

## Ethics statement

The studies involving human participants were reviewed and approved by Ethics Committee of the Institute for Datability Science of Osaka University (July 21, 2020). The patients/participants provided their written informed consent to participate in this study.

## Author contributions

HA, HN, SS, and HT conceived the design and concept of this study. DI, HA, SS, and HT analysed the data, and DI wrote the first draft. TM, VE, NT, MA, TH, YY, and MI provided insightful advices regarding data analysis and interpretation. All authors contributed to the article and approved the submitted version.

## Funding

This work was partially supported by the Innovation Platform for Society 5.0, of the Japan Ministry of Education, Culture, Sports, Science, and Technology (Code: S004541). The funder of the study had no role in data collection, data analysis, data interpretation, writing of the report, or the decision to submit the paper for publication.

## Conflict of interest

The authors declare that the research was conducted in the absence of any commercial or financial relationships that could be construed as a potential conflict of interest.

## Publisher’s note

All claims expressed in this article are solely those of the authors and do not necessarily represent those of their affiliated organizations, or those of the publisher, the editors and the reviewers. Any product that may be evaluated in this article, or claim that may be made by its manufacturer, is not guaranteed or endorsed by the publisher.
